# Ultrasound and multi-biomarker disease activity score for assessing and predicting clinical response to tofacitinib treatment in patients with rheumatoid arthritis

**DOI:** 10.1186/s41927-020-00153-4

**Published:** 2020-10-19

**Authors:** Amir A. Razmjou, Jenny Brook, David Elashoff, Gurjit Kaeley, Soo Choi, Tanaz Kermani, Veena K. Ranganath

**Affiliations:** 1 Department of Medicine, UCLA-David Geffen School of Medicine, Los Angeles, USA; 2 Department of Medicine Statistics Core, UCLA-David Geffen School of Medicine, Los Angeles, USA; 3 Department of Rheumatology, University of Florida Health, Jacksonville, USA; 4 Department of Rheumatology, University of California, San Diego, USA; 5 Department of Rheumatology, UCLA-David Geffen School of Medicine, 1000 Veteran Blvd., RM 32-59, Los Angeles, CA 90095 USA

**Keywords:** Rheumatoid arthritis, Ultrasound, Outcome measures, Multi-biomarker disease activity score

## Abstract

**Background:**

Musculoskeletal ultrasound (MSUS) and the multi-biomarker disease activity (MBDA) score are outcome measures that may aid in the management of rheumatoid arthritis (RA) patients. This study evaluated tofacitinib response by MSUS/MBDA scores and assessed whether baseline MSUS/MBDA scores or their early changes predict later clinical response.

**Methods:**

Twenty-five RA patients treated with tofacitinib were assessed at baseline, 2, 6 and 12-weeks. Power doppler (PDUS) and gray scale (GSUS) ultrasound scores, MBDA score, clinical disease activity index (CDAI), and disease activity score (DAS28) were obtained. Pearson correlations and multiple linear regression models were used to evaluate associations and identify predictors of response to therapy.

**Results:**

MSUS, MBDA scores, CDAI, and DAS28 improved significantly over 12 weeks (*p* < 0.0001). Baseline MSUS and MBDA score correlated with each other, and with 12-week changes in CDAI/DAS28 (r = 0.45–0.62, *p* < 0.05), except for GSUS with DAS28. Two-week change in MSUS correlated significantly with 12-week changes in CDAI/DAS28 (r = 0.42–0.57, *p* < 0.05), except for early change in PDUS with 12-week change in CDAI. Regression analysis demonstrated significant independent associations between baseline PDUS/MBDA score and 6-week change in CDAI/DAS28, with adjustment for baseline CDAI/DAS28 (all *p* < 0.05); and between baseline MBDA scores and 12-week change in DAS28 (*p* = 0.03).

**Conclusions:**

RA patients treated with tofacitinib for 12 weeks demonstrated improvement by clinical, imaging, and biomarker end-points. Baseline PDUS and MBDA score were predictive of CDAI and DAS28 responses. This is the first study to evaluate early measurements of MSUS and MBDA score as predictors of clinical response in RA patients treated with tofacitinib.

**Trial registration:**

ClinicalTrials.gov NCT02321930 (registered 12/22/2014).

## Background

Early treatment and vigilant monitoring are important for preventing joint destruction, impaired function, and poor quality of life in patients with rheumatoid arthritis (RA). Numerous metrics are available to monitor disease activity and inform treatment decisions. Validated RA outcomes include questionnaire-based metrics (Routine Assessment of Patient Index Data 3 [RAPID-3]; Health Assessment Questionnaire [HAQ]), clinical metrics (Clinical Disease Activity Index [CDAI]) and composites of clinical and laboratory-based metrics (e.g., Disease Activity Score [DAS]). While these measures have advanced RA patient care over the last few decades, concerns regarding their subjectivity indicate that objective, sensitive measures are still needed [1, 2].

Musculoskeletal ultrasound (MSUS) and the multi-biomarker disease activity (MBDA) scores are measures that can aid in the management of RA patients. These measures are responsive to RA treatment and may provide additional information beyond that of standard disease activity RA metrics [3, 4]. MSUS assesses synovitis by power Doppler (PDUS) and gray-scale (GSUS) and has been deemed appropriate for use in the management of RA by the American College of Rheumatology (ACR) [5] and the European League Against Rheumatism (EULAR) [3]. MSUS is often more sensitive for detecting synovitis than physical exam alone [6, 7], and has been shown to predict flares of RA [8, 9]. The MBDA score is a validated blood test that measures 12 serum biomarkers for an algorithm that scores RA disease activity on a scale of 1–100. The MBDA score correlates with DAS28-CRP and is a predictor of risk for radiographic progression [10, 11].

Tofacitinib is a small-molecule oral Janus kinase (JAK) inhibitor that is approved for treatment of RA [12]. The efficacy and safety of tofacitinib 5 mg twice daily, with or without conventional synthetic disease modifying antirheumatic drugs (csDMARDs), has been demonstrated in Phase 2 and Phase 3 studies of patients with RA [13–16]. No published studies have assessed MSUS or the MBDA score in RA patients treated with tofacitinib.

Examining the relationship between early changes in MSUS/MBDA scores to later changes in DAS/CDAI is of interest, as it may potentially allow for earlier identification of individuals who benefit from therapy. Additionally, baseline values of MSUS/MBDA scores may aid in differentiating inflammatory disease amenable to treatment, in RA patients with comorbidities where components of DAS/CDAI may be falsely elevated [1, 17]. This study evaluated the response of MSUS (PDUS and GSUS) and the MBDA score in patients treated with tofacitinib, and assessed whether baseline MSUS and MBDA scores or their early changes are predictive of later clinical response as measured by CDAI and DAS28.

## Methods

### Setting

This study was a single-center, open-label, single-arm trial of RA patients treated with oral tofacitinib. The study was conducted at the University of California, Los Angeles (UCLA) Rheumatology Clinics between February 2016 and August 2017. All participants provided written informed consent in person, prior to the conduct of any study procedures. The study was reviewed by the Institutional Review Board (IRB) (IRB# 14–001148) and registered with clinicaltrials.gov (NCT02321930, ID: WI193025).

### Subjects

Inclusion criteria included meeting the ACR/EULAR 2010 RA classification criteria [18], baseline DAS28/ESR ≥ 3.2, baseline total PDUS score ≥ 10 (see below for scoring system), age ≥ 18, and stable ongoing treatment with a conventional synthetic DMARD (csDMARD), such as methotrexate, leflunomide, hydroxychloroquine, sulfasalazine. Exclusion criteria included current use of prednisone > 10 mg per day, prior treatment with tofacitinib, concomitant biologic therapy, active infection, untreated latent tuberculosis, or significant organ dysfunction.

### Study design, treatment, and clinical and laboratory assessments

Study participants were treated with tofacitinib 5 mg orally twice a day for a total of 12 weeks. Patient demographics were collected at the screening visit prior to treatment. Patients were assessed at baseline, 2 weeks, 6 weeks, and 12 weeks. At each visit, patients were assessed by the tender joint count 28 (TJC28), swollen joint count 28 (SJC28), acute phase reactants (high-sensitivity C-reactive protein [hsCRP], erythrocyte sedimentation rate [ESR]), physician and patient global assessments and HAQ. CDAI and DAS28-ESR (DAS28) were determined for each visit. Rheumatoid factor (RF) and anti-cyclic citrullinated peptide antibody (ACPA) were obtained at baseline.

### Musculoskeletal ultrasound

MSUS assessments (PDUS and GSUS) were obtained at screening, baseline, 2 weeks, and 12 weeks by the same sonographer, who had greater than 8 years of experience and is ACR certified in MSUS (RhMSUS certification) (VKR). MSUS assessment was blinded to patient clinical data. A 34-joint US scoring system was used, which assessed bilateral midline wrists (dorsal longitudinal), radio-ulnar joints (dorsal longitudinal/short midline), metacarpophalangeal joints 1–5 (MTP, dorsal long and short, volar longitudinal), proximal interphalangeal joints 2–5 (dorsal/volar longitudinal), metatarsophalangeal joints 2–5 (dorsal longitudinal), and knees (medial parapatellar/lateral parapatellar axial oblique). The MSUS 34-joints were chosen based on commonly affected joints in RA and feasibility of acquiring images, and this protocol has been previously used [1, 19, 20]. MTP1 was excluded as this joint is commonly affected by osteoarthritis. Each image was scored semi-quantitatively on a scale of 0–3 for both PDUS and GSUS. We utilized a modification of the PDUS semiquantitative scoring system proposed by Hammer et al.: 0 = none, 1 = minor, 2 = moderate, and 3 = major degree of PDUS activity [20, 21]. We created an atlas that iteratively improved on the helpful Hammer atlas to allow for improved reliability scores [20]. Each image was also scored for GSUS in the following manner: no synovitis = grade 0, minor synovitis = grade 1, moderate synovitis = grade 2, major synovitis = grade 3. Total PDUS and GSUS scores were calculated as follows: If multiple views of a single joint were assessed, the view with the maximum score was used for the joint, and these 34 scores were summed to represent the total PDUS or GSUS score (range 0–102) [1, 20]. Hereinafter, the total 34-joint PDUS score will be referred to as PDUS, and similarly the total 34-joint GSUS score will be GSUS. A modified version of the German ultrasound 7 score (mUS7) for PDUS and GSUS was also calculated [22]. The mUS7 PDUS and mUS7 GSUS scores do not include a palmar wrist view. Thus, the range of mUS7 PDUS is 0–36 (rather than 0–39), and the range of mUS7 GSUS is 0–24 (rather than 0–27). The same ultrasound machine and transducer (a GE Logic E9 with a 6–15 MHz linear probe; GE Healthcare, Chicago, IL) and standardized settings (red-yellow color map, Doppler frequency: 10.0 MHz, PRF 0.8 KHz, gain adjusted just below noise) were used for each visit and joint for all patients. The same transducer was used for the finger and the knee parapatellar recesses, however, adjustments were made for depth. Ten percent of the images were rescored and compared to the original score. The intra-rater reliability was 0.82 for PDUS and 0.76 for GSUS (weighted Kappa).

### MBDA testing

MBDA scores were determined for all patients at baseline, week 2 and week 12 from serum samples that were shipped on ice within few hours of phlebotomy to the CLIA-certified clinical laboratory of Crescendo Bioscience, Inc., in South San Francisco, CA, for testing. MBDA scores were determined with a validated algorithm based on serum concentrations of 12 protein biomarkers [10]. The biomarkers in the MBDA test reflect the biology of RA and consist of cytokine-related proteins (IL-6, TNF-R1), acute phase reactants (CRP, serum amyloid A), an adhesion molecule (VCAM-1), growth factors (EGF, VEGF-A), matrix metalloproteinases (MMP-1, MMP-3), and adipokines (leptin, resistin). The MBDA score is an integer on a scale of 1 to 100, with disease activity categories of low (< 30), moderate (30–44) and high (> 44) [10]. Minimally clinically important difference (MCID) for MBDA score is ≥8 [23].

### Statistical analysis

The sample size for the study was calculated for the primary analysis of comparing PDUS between baseline and week 12. Based on unpublished data suggesting an effect size for change in PDUS over time of 1.03 (unpublished data), the planned sample size of 25 had 99% power for detecting differences in PDUS over 3 months, assuming a repeated measures ANOVA analysis plan with a 0.05 two-sided level of significance. Repeated measures ANOVA models were used to evaluate changes over time in MSUS, MBDA score and RA disease activity measurements. Each variable was examined for skewness and not found to be substantially skewed. Thus, Pearson correlations were computed for associations between MSUS or MBDA scores and CDAI or DAS28, which were evaluated cross-sectionally and as changes over time. In addition, correlations were calculated between MSUS/MBDA scores and individual components of DAS28/CDAI. Multiple linear regression models for the outcomes of change in CDAI and change in DAS28 were constructed. These models included baseline MSUS or MBDA scores, adjusting for baseline CDAI and DAS28 as appropriate.

## Results

Twenty-five RA patients were enrolled in the trial. Mean age was 52 (SD 9.9) years, mean duration of disease 10.4 years (SD 9.7); 88% of patients were female, 40% Caucasian, 88% were RF and/or ACPA positive (Table 1). At baseline, 72% of patients were receiving a csDMARD, most commonly methotrexate (52%), and 56% were biologic naïve.

**Table 1:** Baseline Patient Characteristics

Total Number of Patients	25
**Age** (years), mean (SD)	52 (9.9)
**Females** (percent)	22 (88%)
**Race** n (%)	
Caucasian	10 (40%)
Black	4 (16%)
Hispanic	5 (20%)
Asian	4 (16%)
Other	2 (8%)
**Seropositive** n (%)	22 (88%)
**Disease Duration** (years), mean (SD)	10.4 (9.7)
**BMI,** mean (SD)	31.0 (10.8)
**Prednisone at Baseline** n (%)	7 (28%)
**csDMARDs at Baseline** n (%)	18 (72%)
Methotrexate	13 (52%)
Sulfasalazine	4 (16%)
Hydroxychloroquine	4 (16%)
Leflunomide	4 (16%)
**bDMARDs before Baseline** n (%)	
TNF inhibitor	8 (32%)
IL-6 inhibitor	1 (4%)
Anti-CTLA4-IgG	2 (8%)
None of the Above	14 (56%)
**Comorbidities** (number), mean (SD)	3.8 (2.6)

*SD* Standard Deviation, *RF* Rheumatoid Factor, *ACPA* Anti-Citrullinated Protein

Antibody; BMI = Body Mass Index; csDMARDs = Conventional Synthetic Disease

Modifying Anti-Rheumatic Drugs; bDMARDs = Biological DMARDs

### Ultrasound, MBDA score, and RA disease activity measures over 12 weeks

**Fig. 1:**
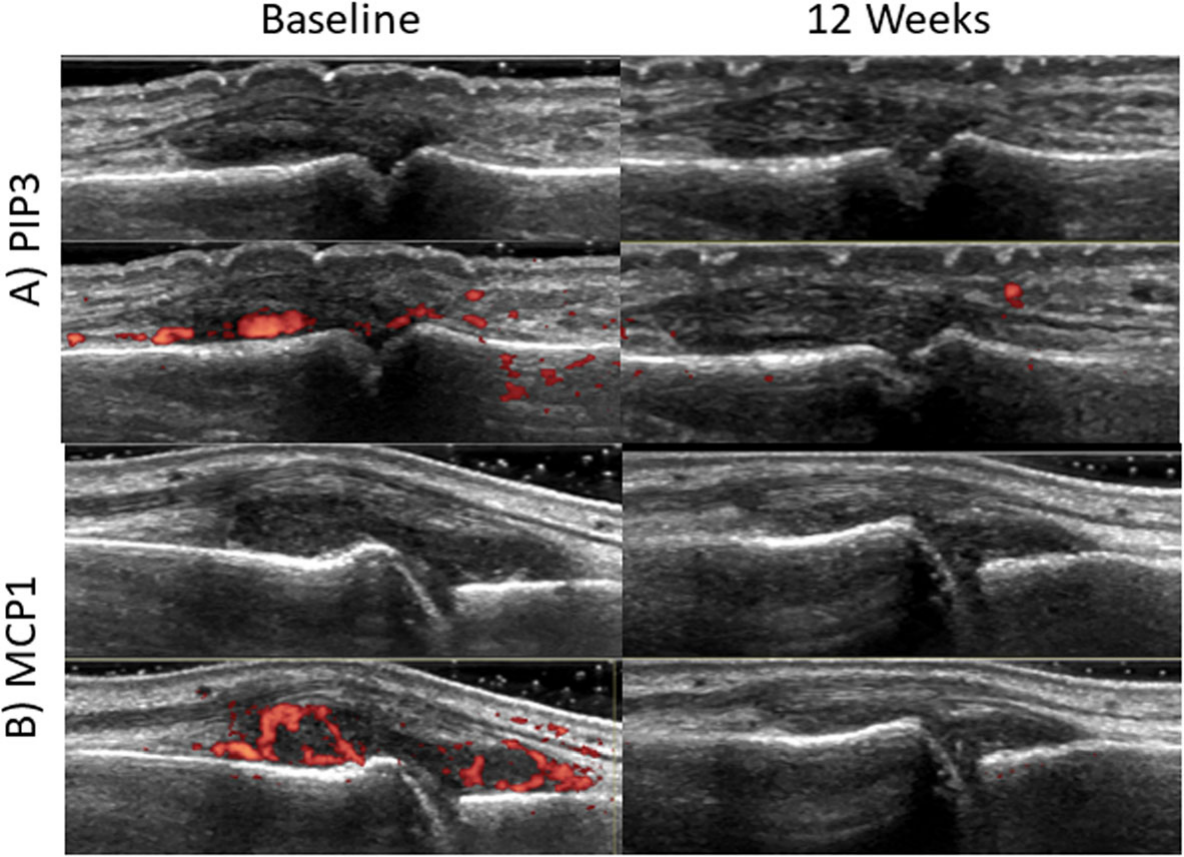
Baseline and 12-Week MSUS images after treatment with tofacitinib (GSUS and PDUS). Panels **a** and **b** show images of PIP3 and MCP1 in both B-mode and power Doppler before and after treatment with tofacitinib

At baseline, patients had high disease activity with a mean CDAI of 39.9 (SD 13.2) and mean DAS28 of 6.3 (SD 1.2) (Table 2). The baseline mean MBDA score was 50.6 (SD 17.5), mean PDUS 28.7 (SD 17.8), mean GSUS was 48.4 (SD 16.5), mean mUS7 PDUS was 10.2 (SD 6.5), and mean mUS7 GSUS was 18.0 (7.1) (Fig. 1). In response to tofacitinib, all of these indices decreased significantly over the 12-week follow-up (*p* < 0.0001) (Table 2). The standardized response means (SRM) for PDUS and mUS7 PDUS (1.18 and 1.10), and GSUS and mUS7 GSUS (0.90 and 0.95) were similar. The MBDA score decreased from baseline to 12 weeks by ≥8 units (i.e., the minimally important difference), in 12 (50%) patients. Twelve-week changes in DAS28/CDAI resulted in the largest standardized response means (SRM) (1.71–1.73), while hsCRP had the smallest SRM (0.36).

**Table 2:** Ultrasound, MBDA Score, and RA Disease Activity Measures Over 12 Weeks

Measure (range/unit)	Baseline mean (SD) *N* = 25	2 weeks mean (SD) *N* = 25	12 weeks mean (SD) *N* = 24	*p*-value	SRM
**PDUS (0–102)**	28.7 (17.8)	19.5 (15.4)	12.2 (10.6)	< 0.0001	1.18
**GSUS (0–102)**	48.4 (16.5)	44.9 (16.4)	37.9 (15.3)	< 0.0001	0.90
**mUS7 PDUS (0–36)**	10.2 (6.5)	7.0 (5.2)	4.1 (4.0)	< 0.0001	1.10
**mUS7 GSUS (0–24)**	18.0 (7.1)	15.7 (7.1)	12.9 (5.9)	< 0.0001	0.95
**MBDA Score (1–100)**	50.6 (17.5)	41.0 (15.1)	39.6 (15.3)	< 0.0001	0.73
**CDAI (0–76)**	39.9 (13.2)	28.6 (13.2)	21.6 (13.0)	< 0.0001	1.73
**DAS28/ESR (0- ~ 9.1)**	6.3 (1.2)	5.2 (1.3)	4.6 (1.4)	< 0.0001	1.71
**TJC28 (0–28)**	12.6 (6.5)	8.7 (6.5)	6.3 (5.8)	< 0.0001	1.12
**SJC28 (0–28)**	13.0 (6.4)	9.3 (6.1)	7.1 (5.4)	< 0.0001	1.26
**Physician Global (0–10)**	6.8 (2.0)	5.4 (1.6)	4.2 (1.6)	< 0.0001	1.53
**Patient Global (0–10)**	7.0 (1.9)	4.9 (2.1)	3.5 (2.6)	< 0.0001	1.31
**ESR (mm/hr)**	39.2 (27.4)	31.6 (22.0)	27.6 (20.3)	0.003	0.55
**hsCRP (mg/L)**	16.7 (20.8)	6.7 (7.4)	8.1 (16.7)	0.02	0.36
**HAQ-DI (0–3)**	1.4 (0.7)	1.0 (0.6)	0.7 (0.6)	< 0.0001	0.83

*PDUS* Power doppler ultrasound, *GSUS* Gray scale ultrasound, *mUS7* Modified ultrasound 7, *MBDA* Multi-biomarker disease activity, *CDAI* Clinical disease activity index, *DAS28/ESR* Disease activity score, erythrocyte sedimentation rate, *TJC* Tender joint count, *SJC* Swollen joint count, *hsCRP* High-sensitivity C-reactive protein, *HAQ-DI* Health assessment questionnaire-disability Index, *SRM* Standardized Response Means. *P*-values represent the significance of the effect of time in repeated measures ANOVA models

### Relationships between ultrasound and MBDA scores

MBDA score demonstrated significant cross-sectional correlations with PDUS at baseline (r = 0.74, *p* < 0.01), 2 weeks (r = 0.61, *p* < 0.05) and 12 weeks (r = 0.50, *p* < 0.05); and with GSUS at baseline (r = 0.61, *p* < 0.01) and 2 weeks (r = 0.41, *p* < 0.05) but not 12 weeks (r = 0.22, *p* > 0.05). These results show that correlations between MBDA score and ultrasound measures were strongest at baseline and remained statistically significant during treatment with tofacitinib.

### Cross-sectional correlations between ultrasound, MBDA score, and RA disease activity outcomes

Cross-sectional analyses demonstrated that PDUS and GSUS correlated significantly with CDAI and DAS28 at all study time points (baseline, 2 weeks and 12 weeks) (Pearson correlations ranging from r = 0.45 to r = 0.65, *p* < 0.05), with the exception of 12-week PDUS with 12-week CDAI (r = 0.38, *p* > 0.05) (Table 3). Both mUS7 PDUS and mUS7 GSUS demonstrated similar correlation with CDAI and DAS28 (r = 0.41–0.58, *p* < 0.05) with the exception of 2-week mUS7 GSUS with 2-week DAS28, and 12-week mUS7 PDUS with 12-week CDAI. The magnitude of these correlations generally decreased over time. MBDA score correlated significantly with DAS28 at baseline (r = 0.47, *p* < 0.05), but not at weeks 2 or 12, and it did not correlate significantly with CDAI at any time point.

**Table 3:** Correlations between MSUS/MBDA Score and Clinical Disease Activity Measures

**Baseline MSUS/MBDA score association with Baseline CDAI/DAS28/ESR**		
	**CDAI Baseline**	**DAS28/ESR Baseline**
**PDUS Baseline**	0.53**	0.58**
**GSUS Baseline**	0.65**	0.53**
**mUS7 PDUS Baseline**	0.45*	0.58**
**mUS7 GSUS Baseline**	0.49*	0.42*
**MBDA Score Baseline**	0.24	0.47*
**2 Week MSUS/MBDA score association with 2 Week CDAI/DAS28**		
	**CDAI 2 Week**	**DAS28/ESR 2 Week**
**PDUS 2 Week**	0.45*	0.49*
**GSUS 2 Week**	0.62**	0.52**
**mUS7 PDUS 2 Week**	0.43*	0.49*
**mUS7 GSUS 2 Week**	0.41*	0.31
**MBDA Score 2 Week**	0.05	0.19
**12 Week MSUS/MBDA score association with 12 Week CDAI/DAS28**		
	**CDAI 12 Week**	**DAS28/ESR 12 Week**
**PDUS 12 Week**	0.38	0.54**
**GSUS 12 Week**	0.52**	0.53**
**mUS7 PDUS 12 Week**	0.28	0.43*
**mUS7 GSUS 12 Week**	0.49*	0.44*
**MBDA Score 12 Week**	−0.19	0.05
**Baseline MSUS/MBDA score association with 12 Week change in CDAI/DAS28**		
	**CDAI (Δ 0–12 weeks)**	**DAS28/ESR (Δ 0–12 weeks)**
**PDUS Baseline**	0.55**	0.45*
**GSUS Baseline**	0.47*	0.25
**mUS7 PDUS Baseline**	0.62**	0.55**
**mUS7 GSUS Baseline**	0.47*	0.33
**MBDA Score Baseline**	0.44*	0.50*
**2 Week change in MSUS/MBDA score association with 12 Week change in CDAI/DAS28**		
	**CDAI (Δ 0–12 weeks)**	**DAS28/ESR (Δ 0–12 weeks)**
**PDUS (Δ 0–2 weeks)**	0.37	0.42*
**GSUS (Δ 0–2 weeks)**	0.44*	0.42*
**mUS7 PDUS (Δ 0–2 weeks)**	0.47*	0.57**
**mUS7 GSUS (Δ 0–2 weeks)**	0.56**	0.51**
**MBDA Score (Δ 0–2 weeks)**	0.22	0.29
**12 Week change in MSUS/MBDA score association with 12 Week change in CDAI/DAS28**		
	**CDAI (Δ 0–12 week)**	**DAS28/ESR (Δ 0–12 week)**
**PDUS (Δ 0–12 week)**	0.58**	0.56**
**GSUS (Δ 0–12 week)**	0.47*	0.34
**mUS7 PDUS (Δ 0–12 week)**	0.60**	0.64**
**mUS7 GSUS (Δ 0–12 week)**	0.54**	0.48*
**MBDA Score (Δ 0–12 week)**	0.44*	0.59**

*p < 0.05, **p < 0.01; *PDUS* Power doppler ultrasound, *GSUS* Gray scale ultrasound, *mUS7* modified ultrasound 7, *MBDA* Multi-biomarker Disease activity score, *CDAI* Clinical disease activity index, *DAS28/ESR* Disease activity score/erythrocyte sedimentation rate

### Relationships between 12-week changes in ultrasound, MBDA score and CDAI and DAS28

Changes in MSUS measures and the MBDA score from baseline to 12 weeks all correlated significantly with changes from baseline to 12 weeks in CDAI and DAS28 (range: r = 0.44 to r = 0.64, *p* < 0.05), with the exception of 12-week changes in GSUS and DAS28 (Table 3).

### Relationships between early measurements of ultrasound/MBDA score and later responses in CDAI/DAS28

Baseline MSUS measures and the MBDA score correlated significantly with changes in CDAI and DAS28 from baseline to week 12 (range: r = 0.45 to r = 0.55, *p* < 0.05), with the exception of baseline GSUS and mUS7 GSUS with 12-week change in DAS28 (Table 3). Changes in MSUS measures from baseline to week 2 correlated significantly with changes from baseline to week 12 in CDAI and DAS28 (range of r = 0.42 to r = 0.44, *p* < 0.05), with the exception of 2-week change in PDUS with 12-week change in CDAI (Table 3). Change in MBDA score from baseline to week 2 did not correlate significantly with 12-week changes in CDAI or DAS28.

### Relationships between ultrasound, MBDA score, and individual components of CDAI and DAS28 (TJC28, SJC28, MD global, patient global, ESR)

Cross-sectionally, MSUS measures correlated with SJC28 at baseline, and week 2 (r = 0.43 to 0.76, *p* < 0.05). MSUS measures and the MBDA score correlated well with ESR at baseline (r = 0.50 to 0.74, *p* < 0.05). None of the MSUS measures or the MBDA score correlated with TJC28 at any time point.

### Multiple linear regression for associations between baseline ultrasound score and changes in CDAI and DAS28 from baseline to weeks 6 or 12

Multiple linear regression demonstrated a positive independent association between baseline PDUS and change in DAS28 from baseline to week 6 (*p* = 0.01), with adjustment for baseline DAS28. Similarly, baseline PDUS was independently associated with change in CDAI from baseline to week 6 (*p* = 0.004), with adjustment for baseline CDAI. Baseline PDUS showed a trend toward positive association with changes in CDAI and DAS28 from baseline to week 12, (*p* = 0.07 and *p* = 0.05, respectively). By contrast, GSUS was not associated with CDAI or with DAS28 responses from baseline to week 6 or week 12. The baseline CDAI and baseline DAS28 were not significantly associated with the 6-week or 12-week responses to therapy for CDAI or DAS28.

### Multiple linear regression for associations between baseline MBDA score and 6-week and 12-week changes in CDAI and DAS28

Multiple linear regression demonstrated a positive independent association between baseline MBDA score and change in CDAI or DAS28 from baseline to week 6 (both *p* = 0.006), with adjustment for baseline CDAI and DAS28, respectively. Baseline MBDA score was associated with 12-week change in DAS28 (*p* = 0.03), with adjustment for baseline DAS28, with a trend toward association with 12-week change in CDAI (*p* = 0.08), with adjustment for baseline CDAI.

### Safety

There were two reports of adverse events, both moderate in severity. One patient had a tendon rupture of unclear etiology and the other had an upper respiratory infection. Another patient was withdrawn from the study after the week 2 visit, due to the development of a breast mass during the trial, with subsequent imaging demonstrating a simple cyst. In addition, one patient was diagnosed with diffuse large B-cell lymphoma approximately 2 months after conclusion of the trial.

## Discussion

This study investigated the response of RA patients treated with tofacitinib using traditional clinical measures (CDAI and DAS28), and imaging and biomarker measures (MSUS and MBDA score). Our data is consistent with other studies of tofacitinib which demonstrated significant responses by CDAI and DAS28 [15, 16, 24] in patients with RA. This study is the first to demonstrate significant improvements in MSUS measures (with both 34-joint, and mUS7 methods)and MBDA scores in response to tofacitinib. Moreover, it demonstrated that PDUS and MBDA score were correlated with each other and that, at baseline, each predicted later responses of CDAI and DAS28.

Several studies have demonstrated the ability of MSUS to monitor treatment response and predict radiographic progression in patients with RA, including patients in clinical remission [9, 25–28]. Fewer studies have evaluated baseline MSUS and its early changes for predicting later clinical response to treatment [29–31]. Kawashiri and colleagues evaluated 39 RA patients treated with biologic DMARDs over 24 weeks. They found that the percentage decrease of GSUS and PDUS over 12 weeks was greater for those patients with EULAR moderate and good responses compared to non-responders at 24 weeks (*p* < 0.05) [31]. Ellegaard et al. demonstrated in a cohort of 109 RA patients that baseline PDUS predicted persistence of anti-TNF-alpha therapy whereas conventional clinical measures at baseline did not [29]. In a study of 25 biologic-naïve RA patients treated with abatacept, we previously found that baseline PDUS, measured using the US7 ultrasound protocol [22], significantly correlated with 52-week change in DAS28 [30]. In addition, there was a trend toward the association of PDUS with 52-week change in DAS28 after adjustment for baseline DAS28 (0.06).

The present tofacitinib study found that higher baseline PDUS values were associated with larger CDAI and DAS28 responses at 6 weeks, with a trend observed at 12 weeks. Our study is distinct among imaging studies in that we included baseline clinical disease activity measures in the multiple regression analyses. Additionally, we found that early changes in PDUS,GSUS, and mUS7 scores, i.e., from baseline to 2 weeks, showed a significant correlation with 12-week changes in CDAI and DAS28.

Prior studies have reported that baseline MBDA score can predict risk for radiographic progression [11, 32–34], and that the MBDA score is a better predictor of radiographic progression than conventional measures of RA disease activity [33, 35]. A study of patients with early RA found that lower MBDA scores in patients with inadequate clinical response to methotrexate monotherapy were associated with better clinical responses to subsequent triple therapy [36]. Another study of patients with early RA - treated with MTX alone or with adalimumab - found that, for each treatment, baseline MBDA score and change in MBDA score from baseline to 3 months were associated with DAS28 improvement from baseline to 6 months [31]. Our study, of tofacitinib treatment for patients with established RA, showed that baseline MBDA score correlated significantly with changes in CDAI and DAS28 from baseline to week 12, although change in MBDA score, from baseline to week 2, did not correlate with the later changes. The MBDA score significantly correlated with PDUS at all timepoints, with the strongest correlation found at baseline (r = 0.74), suggesting that there may be biologic differences in therapeutic responsiveness of these measures.

The MSUS and MBDA scores, at baseline and their early changes, may be of clinical value for predicting outcomes in patients started on treatment with tofacitinib. In this RA cohort with high disease activity, MSUS/MBDA scores correlated fairly well with the clinical measures of CDAI/DAS28. In a more heterogeneous population (e.g., with obesity, hand osteoarthritis, fibromyalgia), PDUS and MBDA score may help in the assessment of RA disease activity and stratify patients according to predicted response. If baseline measures or their early changes suggest possible treatment failure, an earlier change of treatment could be considered, potentially improving the clinical course and minimizing unnecessary healthcare utilization. In addition, elevated baseline MSUS and/or MBDA scores may provide value as inclusion criteria in randomized clinical trials allowing for a more homogenous group of RA patients with high disease activity and risk for joint damage [37].

Interestingly, when assessing the correlations of MSUS measures and the MBDA score with the individual components of the CDAI and DAS28 (TJC28, SJC28, MD Global, Patient Global, ESR), we found no significant correlations with TJC28 across any time point. In their review, Edwards et al. found that pain catastrophizing, or as they defined “the tendency to ruminate about and magnify pain”, and depression were frequently observed in patients with rheumatologic conditions, and adversely affect several outcomes [38]. In a prospective trial of RA patients starting bDMARDs, Hammer et al. found that pain catastrophizing was associated with patient reported outcomes and the disease activity scores which incorporated them; however, there was no correlation with MSUS [2]. Data such as this suggests a complex interplay between the perception of pain, patient reported outcomes, and inflammatory disease. Further research is warranted.

Limitations of this study pertain to its sample size of twenty-five patients. Nevertheless, we did demonstrate significance in the primary end-points. The small sample size limited our ability to add other covariates into the linear regression model. The patient cohort had high baseline clinical disease activity (CDAI 39.9, DAS28 6.23), as well as long disease duration (10.4 years), which may limit the applicability of the data to other patient populations.

## Conclusions

The results of this open-label trial suggest that baseline PDUS and MBDA scores correlated with each other, tracked response to treatment with tofacitinib and, at baseline, predicted 6-week and 12-week clinical responses to therapy. In addition, the 2-week change in PDUS was found to be predictive of 12-week DAS28 response. Future studies could aim to further elucidate the utility of MSUS and the MBDA score in RA management, and investigate how early changes in these measures may help provide individualized RA patient care.

## Data Availability

The datasets used and/or analyzed during the current study are available from the corresponding author on reasonable request.

## Abbreviations

RA: Rheumatoid Arthritis
RAPID-3: Routine Assessment of Patient Index Data 3
HAQ: Health Assessment Questionnaire
CDAI: Clinical Disease Activity Index
DAS: Disease Activity Score
MSUS: Musculoskeletal Ultrasound
MBDA: Multi-biomarker Disease Activity
PDUS: Power Doppler Ultrasound
GSUS: Gray Scale Ultrasound
ACR: American College of Rheumatology
EULAR: European League Against Rheumatism
csDMARDs: Conventional Synthetic Disease Modifying Antirheumatic Drugs
TJC28: Tender Joint Count 28
SJC28: Swollen Joint Count 28
hsCRP: High-sensitivity C-reactive Protein
ESR: Erythrocyte Sedimentation Rate
RF: Rheumatoid Factor
ACPA: Anti-cyclic Citrullinated Peptide Antibody
MCID: Minimal Clinically Important Difference
